# Bilateral Retinal Detachments Associated with Inversion Table Therapy

**DOI:** 10.7759/cureus.1098

**Published:** 2017-03-15

**Authors:** Valerie C Lerebours, Austin J Rohl, Saad Shaikh

**Affiliations:** 1 Ophthalmology, Howard University College of Medicine; 2 University of Central Florida College of Medicine; 3 Ophthalmology, University of Central Florida College of Medicine

**Keywords:** inversion therapy, inversion table, vitreoretinal, detachment, retinal detachment, retinal tear, gravity

## Abstract

To report a case of sequential bilateral inferior retinal detachments secondary to inversion table therapy.

A 67-year-old-male developed inferior rhegmatoegnous retinal detachments (RRD) in both eyes on two different occasions with the use of inversion therapeutic tables.

Various predisposing factors have been documented for RRD such as previous cataract surgery, peripheral retinal degenerations, high myopia, history of previous retinal detachments and direct ocular trauma. The authors report here a case of inferior retinal detachments associated with the use of inversion therapy. Physical therapists, physical medicine rehabilitation physicians, and retinal specialists need be aware of this potential complication.

## Introduction

The development of a vision-threatening rhegmatogenous retinal detachment (RRD) is initiated by a peripheral retinal break [1]. The most common location for rhegmatogenous retinal detachments is superior [2]. Various predisposing factors have been documented for RRD such as previous cataract surgery, peripheral retinal degenerations, high myopia, history of previous retinal detachments, and direct ocular trauma. In the following, we report a case of bilateral inferior retinal detachments associated with inversion therapy for back pain. Informed consent was obtained for this study.

## Case presentation

A 67-year-old male with a medical history of hypertension and heart failure presented to our clinic with an ocular complaint of floaters and superotemporal visual field loss in his right eye for three days. His ocular history was significant for a diagnosis of pigment dispersion glaucoma for which he was being treated with topical intraocular pressure (IOP) lowering agents and multiple bilateral blepharoplasty procedures. He was mildly myopic with a refraction of -0.75 -2.00 @ 055 in the right eye and -0.75 -2.25 @ 090 in the left eye. His visual acuity was 20/70 in the right eye and 20/20 in the left eye with correction. Ocular examination revealed multiple iris transillumination defects, mild age-related nuclear sclerotic cataracts and open angles with pigmentation in the trabecular meshwork by gonioscopy and normal intraocular pressure (IOP) bilaterally. At this time, the patient was not on IOP-lowering medications. Funduscopic examination of the right eye revealed a cup-to-disc ratio of 0.6 and a macula-off retinal detachment from five to ten o’clock, secondary to a retinal break adjacent to an area of pigmented lattice degeneration at seven o’clock. Fundus examination of the left eye demonstrated inferior chorioretinal and pigmentary changes, a cup-to-disc ratio of 0.4, but otherwise normal findings. The patient underwent an uncomplicated retinal detachment repair by vitrectomy, gas-fluid exchange and endolaser therapy, followed by cataract extraction with intraocular lens placement in both eyes six months later. 

Eleven months after the onset of a retinal detachment in the right eye, the patient presented with a “dark curtain” and flashes involving the superior visual field of his left eye. His visual acuity was 20/30 and the intraocular pressure (IOP) was normal in both eyes. Over the proceeding months, he had been placed on multiple IOP lower medications due to the progression of his glaucoma, left greater than right, by his primary eye care provider. He was taking brimonidine and travoprost in both eyes. Other than the presence of well-positioned intraocular lenses bilaterally, the anterior segment examination was unchanged from the previous year. Funduscopic examination of the left eye demonstrated the progression of his glaucomatous optic neuropathy (cup-to-disc ratio of 0.6) with a macular splitting retinal detachment from three to eight o’clock associated with multiple retinal tears. There were two retinal tears anterior to the equator at four o’clock and six o’clock and an additional retinal tear at seven o’clock immediately posterior to the equator adjacent to the inferonasal retinal vessels. Examination of the right eye demonstrated an attached retina, pigmented laser scars for 360 degrees and a cup-to-disc ratio of 0.6. On further questioning, the patient indicated that he had been utilizing inversion table therapy several times a month, for several minutes at a time, over the previous seven years for back pain. Again, he underwent uncomplicated retinal detachment repair by vitrectomy, gas-fluid exchange, and endolaser therapy. He was advised to discontinue all future inversion table therapy.

**Figure 1:**
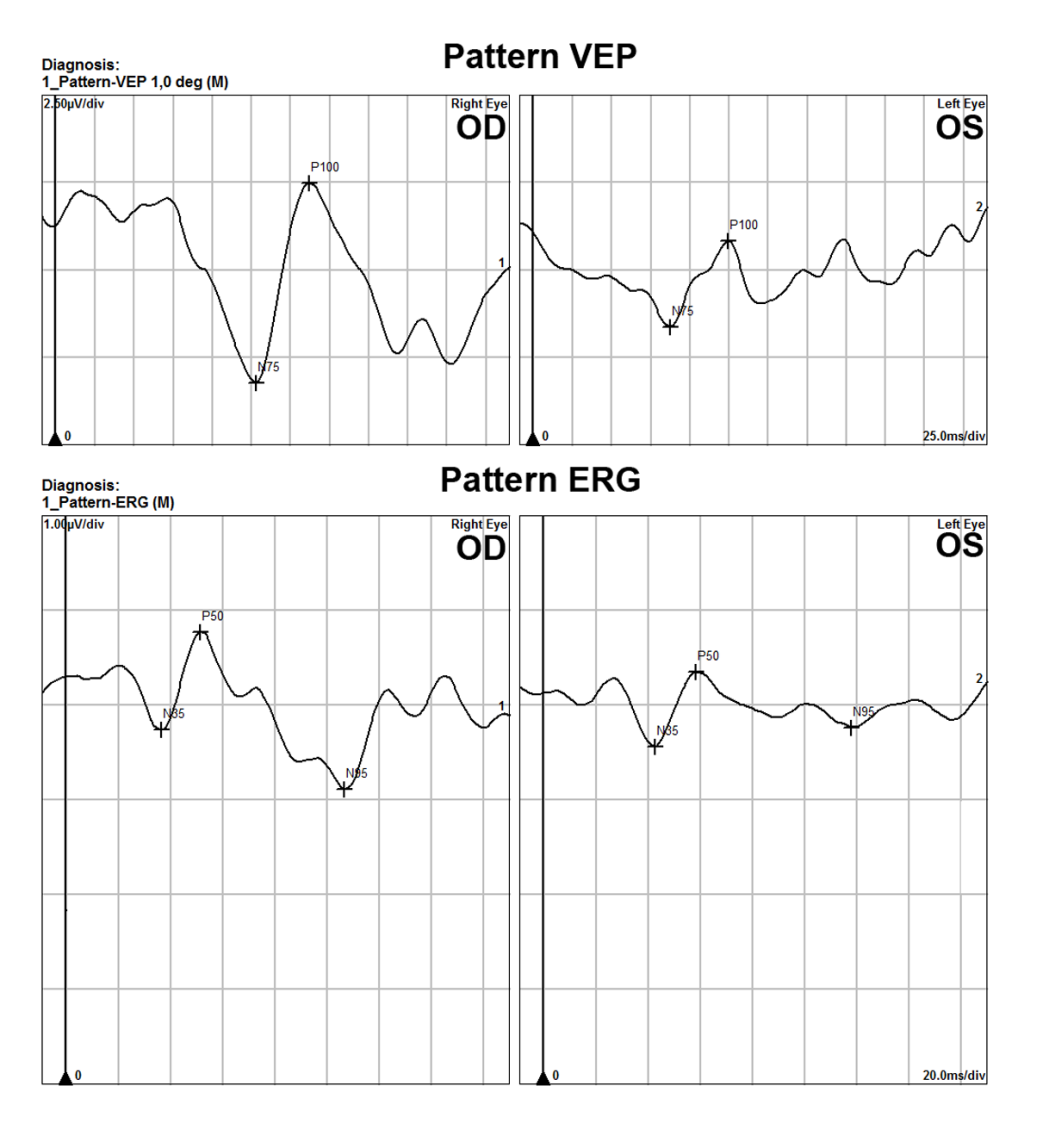
Pattern visual evoked potential (VEP) and pattern electroretinography (ERG) Pattern VEP demonstrates P100 amplitude decrease in the left eye. Pattern ERG of the left eye also demonstrates decreased N95 amplitudes consistent with optic nerve damage. Values in the right eye were low for laboratory normals as well

Over the subsequent eight months, progressive visual field loss and cupping were noted in the left eye, despite good IOP control. His retinae remained attached bilaterally. Funduscopic examination demonstrated pigmented laser scars in the inferior periphery of the right eye and for 360 degrees throughout the periphery and encircling the posterior inferonasal break in the left eye. Optic nerve pallor was noted along with a cup-to-disc ratio of 0.8 in the left eye. An afferent pupillary defect had developed in the left eye. Electrophysiology testing was performed and demonstrated an abnormal pattern electroretinogram (ERG) and pattern visual evoked potential (VEP) in the left eye with otherwise normal full field ERG’s bilaterally consistent with optic nerve damage in the left eye (Figure 1). 

## Discussion

Inversion therapy is a form of spinal traction that uses a tilting board where the person is secured and angled downwards. This provides less tension on the spine and relieves back pain [3]. There has been a single previous case report, over thirty years ago, documenting an inferior retinal detachment in the setting of inversion therapy [4]. In the reported case, gravity boots were used which subjected the patient to a comparatively more severe 180-degree tilt and the full effect of gravity, as compared to the modern form of inversion therapy which utilizes a tilt table.

Our patient developed retinal detachments associated with inferior retinal breaks in both eyes. Although he was mildly myopic and previously had cataract surgery in his left eye, he had no other identifiable risk factors for developing a retinal detachment. The sequential nature of the detachments and the inferior location of the pathology suggests that inversion therapy had more than a circumstantial role in the onset of pathology. The pathogenesis of an inferior rhegmatogenous retinal detachment secondary to the use of inversion therapy is likely due to gravity-induced vitreous traction on the inferior retina resulting in a tear [4]. Inversion positions such as headstands, which are very common in the practice of yoga, are known to cause ocular manifestations such as central retinal vein occlusion, an increase in intraocular pressure and progression of glaucoma [5-6]. Our patient was also found to have developed optic neuropathy in his left eye further complicating his clinical course. He demonstrated glaucomatous progression involving the same eye before the onset of his retinal detachment. He was not, however, on miotic glaucoma therapy, such as pilocarpine, which has an associated risk of retinal detachments. Therefore, it is not clear what role exactly inversion table therapy; the retinal detachment procedure itself, glaucomatous progression, and/or an additional ischemic optic nerve event might have had in the poor visual outcome of the left eye. Ocular complications from inversion therapy can be sight threatening. Educating our patients on the potential ocular risks associated with the use of inversion therapy is imperative.

## Conclusions

Inversion therapy can be associated with the development of inferior retinal detachments. Physical therapists, physical medicine rehabilitation physicians, and retinal specialists need be aware of this potential complication associated with the use of inversion tables.
